# Dynamics and implications of anti-drug antibodies against adalimumab using ultra-sensitive and highly drug-tolerant assays

**DOI:** 10.3389/fimmu.2024.1429544

**Published:** 2024-08-22

**Authors:** Xiaoliang Ding, Ling Xue, Mingjun Wang, Shengxiong Zhu, Kouzhu Zhu, Sheng Jiang, Jian Wu, Liyan Miao

**Affiliations:** ^1^ Department of Pharmacy, The First Affiliated Hospital of Soochow University, Suzhou, China; ^2^ Institute for Interdisciplinary Drug Research and Translational Sciences, Soochow University, Suzhou, China; ^3^ Department of Rheumatology, The First Affiliated Hospital of Soochow University, Suzhou, China; ^4^ College of Pharmaceutical Sciences, Soochow University, Suzhou, China

**Keywords:** adalimumab, anti-drug antibodies, electrochemiluminescence, immunogenicity, neutralizing antibodies

## Abstract

**Background:**

Adalimumab induces the production of anti-drug antibodies (ADA) that may lead to reduced drug concentration and loss-of-response, posing significant clinical challenges. However, traditional immunoassays have limitations in terms of sensitivity and drug-tolerance, hindering the insights of ADA response.

**Methods:**

Herein, we developed an integrated immunoassay platform combining the electrochemiluminescence immunoassay with immunomagnetic separation strategy. A longitudinal cohort study involving 49 patients with ankylosing spondylitis was carried out to analyze the dynamic profiles of ADA and to investigate the impact of ADA on adalimumab pharmacokinetics using a population pharmacokinetic model. Additionally, cross-sectional data from 12 patients were collected to validate the correlation between ADA levels and disease relapse.

**Results:**

The ADA assay demonstrated high sensitivity (0.4 ng/mL) and drug-tolerance (100 μg/mL), while the neutralizing antibodies (NAB) assay showed a sensitivity of 100 ng/mL and drug-tolerance of 20 μg/mL. Analysis of the longitudinal cohort revealed that a majority of patients (44/49, 90%) developed persistent ADA within the first 24 weeks of treatment. ADA levels tended to plateau over time after an initial increase during the early immune response phase. Further, nearly all of the tested patients (26/27, 96%) were classified as NAB positive, with a strong correlation between ADA levels and neutralization capacity (R^2^ = 0.83, *P* < 0.001). Population pharmacokinetic modeling revealed a significant positive association between model-estimated individual clearance and observed ADA levels. Higher ADA levels were associated with adalimumab clearance and disease relapse in a cross-sectional cohort, suggesting a promising ADA threshold of 10 for potential clinical application. Moreover, the IgG class was the primary contributor to ADA against adalimumab and the apparent affinity exhibited an increasing trend over time, indicating a T-cell dependent mechanism for ADA elicitation by adalimumab.

**Conclusion:**

In summary, this integrated immunoassay platform shows promise for in-depth analysis of ADA against biologics, offering fresh insights into immunogenicity and its clinical implications.

## 1 Introduction

Monoclonal antibody-based biopharmaceuticals have significantly advanced current therapies for cancer and autoimmune diseases. However, a major concern with long-term clinical use is the immunogenicity elicited by repeated drug administration (1). The host immune system recognizes the differing relevant epitopes in the biologic drug as foreign and then triggers the specific anti-drug antibodies (ADA) against it, leading to the formation of drug–ADA immune complexes and accelerated drug clearance. Neutralizing antibodies (NAB), a subset of ADA, have the ability to directly block the drug from binding to its target, thereby neutralizing its pharmacological activity. The development of ADA and NAB can potentially impact drug pharmacokinetics, pharmacodynamics and safety.

Adalimumab has been approved for the treatment of various inflammation-mediated diseases, including inflammatory bowel diseases, rheumatoid arthritis, ankylosing spondylitis, and psoriasis. Its high specificity for the target and strong binding to tumor necrosis factor α (TNF-α) have made it the most widely prescribed biological agent globally for the past decade. Initially, adalimumab was perceived as less immunogenic since it is a fully human monoclonal antibody derived from phage display technology. However, recent advancements in bioanalysis have revealed that the prevalence and impact of ADA formation were previously underestimated (2). Numerous studies have explored the relationships between ADA formation, drug concentrations, and treatment effectiveness (3–5).

Nevertheless, there are major gaps in the knowledge on the characteristics of ADA against adalimumab, which hinder a comprehensive understanding of immunogenicity and its clinical implications. For example, limited data exists on the dynamic profile of ADA against adalimumab, including onset time, response duration and magnitude evolution following initial and subsequent doses. Critical information is missing about the threshold relevant to adalimumab pharmacokinetics and treatment effectiveness. Moreover, the kinetics of NAB development and its correlation with ADA are poorly understood. Additionally, detailed features of ADA against adalimumab, such as isotype switching and affinity maturation, remain unclear. Utilizing immunoassays with high sensitivity and drug-tolerance is expected to help fill these knowledge gaps. Existing knowledge on ADA against adalimumab has mainly been obtained using drug-sensitive assays, like traditional bridging ELISA and radioimmunoassay, which can only detect ADA in the presence of low concentrations or absence of the drug (6, 7). Currently, the electrochemiluminescence (ECL) platform is now widely used in the pharmaceutical industry due to its drug-tolerance and sensitivity (8). However, the bridging-ECL immunoassay format may not effectively capture IgG4 and IgM class ADA due to the nature of monovalent antibodies of IgG4 and weak affinity of IgM. NAB are typically detected using cell-based assays or competitive immunoassays (9, 10), but challenges related to sensitivity and drug-tolerance hinder the accurate detection and interpretation of NAB data. Hence, there is a need for immunoassays with high sensitivity, drug-tolerance, and the ability to recognize all ADA isotypes to fully comprehend the landscape of ADA against adalimumab.

Herein, an integrated analytical platform was developed to comprehensively evaluate ADA against adalimumab. This platform utilizes immunomagnetic separation in combination with the ECL detection system, enhancing the drug-tolerance and sensitivity. A longitudinal cohort study was conducted to analyze the dynamics of ADA and NAB levels and their quantitative impact on adalimumab pharmacokinetics. Furthermore, a cross-sectional cohort study investigated the relationship between ADA levels and disease relapse. Additionally, the analytical platform was used to explore class switching and affinity maturation of enriched ADA. These methodologies provide valuable insights into the mechanisms underlying ADA formation and improve the clinical applicability of ADA data in patient care.

## 2 Materials and methods

### 2.1 Patients

Two distinct groups of patients were enrolled in this study, each with access to different types of data. The first group consisted of forty-nine patients with ankylosing spondylitis who were either initiating or continuing adalimumab therapy. This prospective observational single-center cohort study was conducted between September 2021 and May 2023 at the Department of Rheumatology, the First Affiliated Hospital of Soochow University (Suzhou, China). The patients included in the study were either biologically naïve or had prior experience with biological treatment for at least 6 months. None of the patients received concomitant immunomodulatory drugs. Longitudinal blood samples were collected at baseline and prior to each subsequent adalimumab injection randomly in a clinical setting.

To investigate the relationships between adalimumab levels, magnitude of ADA and disease relapse, we analyzed cross-sectional therapeutic drug monitoring data and retrospective electronic medical records from a separate set of 12 patients who had been receiving adalimumab maintenance therapy for more than 3 months. This group comprised 8 patients with Crohn’s disease and 4 patients with ankylosing spondylitis. Among these patients, four experienced disease flare leading to drug discontinuation, while clinical remission was maintained in eight patients as evaluated by a physician using disease activity scores or clinical symptoms (Crohn’s Disease Activity Index for Crohn’s disease, Ankylosing Spondylitis Disease Activity Score for ankylosing spondylitis).

All protocols were approved by the Institutional Review Board of the First Affiliated Hospital of Soochow University (No. 2021–078), and all patients provided written informed consent.

### 2.2 Immunomagnetic separation of ADA

To isolate ADA from plasma while minimizing carryover of the residual drug or biotinylated drug leaching, magnetic beads covalently crosslinked with adalimumab (25 μg protein/mg beads) were prepared according to the manufacturer’s instructions (BeaverBio, BeaverBeads Mag NHS Kit, 300 nm) prior to affinity separation. Before use, glycine buffer (100 mM, pH 2.0) was added to wash the beads, and then the beads were washed three times with PBST (0.1% Tween in phosphate buffer solution) using a magnetic rack. Beads covalently coupled with adalimumab were finally resuspended in Tris buffer (1.5 M, pH 9.6).

Ten-microliter plasma samples were diluted with 300 mM acetic acid at a ratio of 1:9 on a shallow 96-well plate, followed by incubation for 15–20 min with shaking at room temperature. After the ADA-drug complexes were dissociated, 0.1 mg of beads/24 μL buffer were added to each acidified sample to capture ADA at room temperature for 1 h at 1200 rpm. After incubation, the beads were washed three times with PBST using a magnetic separator. Finally, 100 μL of glycine buffer (100 mM, pH 2.0) was added to each sample to elute the ADA from the beads, and the mixture was shaken for 15–20 min at 1200 rpm. The elution supernatant was transferred for subsequent analysis.

### 2.3 Measurement of ADA and NAB with the Meso Scale Discovery (MSD®) platform

An ECL technique based on the MSD platform was used to measure ADA and NAB against adalimumab after ADA purification. Pooled biological-naïve human plasma (n = 20) was used as the negative control (NC). NC samples spiked with rabbit-anti-adalimumab idiotype polyclonal antibodies (pAb, made by Abcepta Biotech) were prepared to assess the performance of the ADA assay as positive control (PC), and NC samples spiked with an anti-idiotype antibody against adalimumab (Bio-Rad, AbD18655_hIgG1, catalogue no. HCA204) were used as PC samples for the NAB assay.

The ADA assay was configured in direct ligand binding format. In brief, the eluted supernatant was directly coated on an MSD high-bind plate, which was incubated for 1 h at 37°C with shaking at 500 rpm. Then, the plate was washed using a microplate washer (BioTek, 405LS) and blocked with 1% BSA in PBS for 1 h at 37°C with shaking at 500 rpm. The plate-bound ADA was detected with ruthenium-labeled adalimumab. After the final incubation step and washing, 2X Read Buffer was added, and the ECL signal of the plate was read on a QuickPlex SQ120 Reader (MSD). The ECL signal was proportional to the ADA level, and each result was converted to a signal-to-NC ratio (S/N). The assay was validated according to white paper (11).

For the NAB assay, a competitive ligand binding (CLB) assay in drug capture format was used to evaluate the neutralization capacity of the ADA. As ruthenium-labeled TNF-α would be unable to bind the adalimumab pre-coated on the plate if the ADA had neutralization capacity, a high ADA neutralization capacity would result in a reduction in the ECL signal. Thirty microliters (5 ng/mL in PBS) of biotin-labeled adalimumab was coated on the MSD streptavidin plate for 1 h at room temperature, followed by the addition of 50 μL of ADA supernatant and 7 μL of neutralization buffer (1 M Tris buffer, pH 8.8). After incubation, the residual adalimumab was detected by ruthenium-labeled TNF-α (100 ng/mL in PBS) and the ECL signal of the plate was read on a QuickPlex SQ120 Reader (MSD) by adding 2X Read Buffer. The sample ECL signal relative to the blank ECL signal (B/B0) reflected the neutralization capacity. The assay was validated according to white paper (12).

### 2.4 Measurement of IgG, IgM or IgA class antibodies to adalimumab

Adalimumab-Fab was prepared to mitigate interference with the IgG detection procedure. Adalimumab was digested by papain to produce specific Fab fragments of adalimumab, according to the manufacturer’s instructions (Pierce™ Fab Preparation Kit, Thermo Scientific). MSD high-binding plate was coated overnight with adalimumab-Fab (1 μg/mL in PBS). After washing and blocking, 50 μL of ADA supernatant and 7 μL of neutralization buffer (1 M Tris buffer, pH 8.8) were added and the plate was incubated at 37°C for 60 min with shaking at 500 rpm. For detection, ruthenium-labeled anti-human IgG (GenScript, catalogue no. V90401), anti-human IgM (GenScript, catalogue no. A02128) antibody, or anti-human IgA antibody (Merck, Rabbit monoclonal, catalogue no. SAB5600221) with ruthenium-labeled anti-rabbit antibody (Abcepta Biotech) was added, and the ECL signal of the plate was read on a QuickPlex SQ120 Reader (MSD). The positive cut-off signal was calculated based on the mean ECL signal obtained with a panel of 10 adalimumab naïve samples plus 3 standard deviations.

### 2.5 Solution equilibrium titration for apparent affinity estimation

In brief, a fixed concentration of enriched ADA was incubated with a series of concentrations of biotin-labeled adalimumab (1–10000 pM) until equilibrium was reached, and then the ADA-biotin-labeled adalimumab complexes were removed using streptavidin-beads (Beaver, BeaverBeads Streptavidin, 1 μm) under a magnetic rack. The unbound ADA that remained in solution were measured using a direct ADA assay, and a kinetic equation was fitted to determine K_D_ value using the custom program in GraphPad (Prism 8, La Jolla, CA). The equation was as follows (13):


$$\text{ECL signal}={\text{ECL}}_{max}\left(1-\frac{1}{{\left(\frac{K_{D}}{Dose}+1\right)}^{2}}\right)$$

where ECL_max_ is the maximum signal when no drug is added, K_D_ is the apparent equilibrium dissociation constant, Dose is the amount of biotin-labeled adalimumab added to the enriched ADA.

### 2.6 Measurement of adalimumab levels

The plasma adalimumab concentration was quantified using a validated sandwich ELISA. Microtiter plates (Thermo, catalogue no. 446469) were coated with an anti-idiotype antibody, a mouse monoclonal antibody specific to adalimumab (GenScript, catalogue no. A01954). The bound adalimumab was then detected using biotinylated mouse anti-adalimumab antibody (GenScript, catalogue no. A01956). Horseradish peroxidase-labeled streptavidin (Solarbio, catalogue no. SE068) and 1-Step Ultra TMB-ELISA substrate solution (Thermo, catalogue no. 34028) were consecutively added to the plate to generate a chromophore, and the color development was stopped by adding a 2 N H_2_SO_4_ solution. The colorimetric intensity was determined by a microplate reader (Thermo, Multiskan Go) at 450 nm with correction based on the signal at 630 nm.

### 2.7 Population pharmacokinetic analysis

Population pharmacokinetic analysis was performed by NONMEN (version 7.5.0; ICON Development Solutions) with Wing for NONMEM (version 750), R (version 3.5.2) and the Pirana interface (version 2.9.4, Certara).

A one-compartment pharmacokinetic model with first-order absorption and elimination (ADVAN2 and TRANS2) was selected to describe the pharmacokinetics of adalimumab based on published data (14). The apparent clearance (CL/F) and apparent volume of distribution (V/F) were estimated, while the absorption rate constant (KA) was fixed due to the limited sampling time points. All parameters were estimated with the first-order conditional estimation with interaction (FOCE-I) algorithm.

The interindividual variability (IIV) of the parameters was modeled using an exponential model as follows:


$${\text{P}}_{ij}=\text{TVP}\times \text{exp}\left({\text{η}}_{ij}\right)$$

where P*_ij_* represents the *i*-th individual value of the parameter on the *j*-th occasion, TVP represents the typical population value of the parameter, and η represents the interindividual variability of the pharmacokinetic parameter and is normally distributed with a mean of 0 and a variance of ω^2^.

The residual error of the model using a combined (proportional plus additive) model was calculated as follows:


$$\text{Y}=\text{CONC}+\text{sqrt}\left({\text{CONC}}^{2}\times {\text{θ}}_{PROP}^{2}+{\text{θ}}_{ADD}^{2}\right)\times \text{ϵ}$$

where θ*_PROP_* represents the parameter of the proportion residual error, θ*_ADD_* represents the parameter of the additional residual error, CONC represents the individual predicted adalimumab concentration, Y represents the observed value, and ε represents the residual error and is assumed to be normally distributed with a mean of 0 and variance of σ^2^.

Based on prior knowledge, normal fat mass (NFM) and ADA levels were introduced into the model using the following general equation:


$$\text{θ}={\text{θ}}_{1}\times {\left(\frac{\text{Covariate}}{\text{mean}}\right)}^{{\text{θ}}_{2}}$$

where θ_1_ is the population estimate of the parameter, Covariate is the continuous covariate, mean is the average of the continuous covariate, and θ_2_ is the estimated coefficient of the continuous covariate. The NFM was calculated based on our previously published model (15). These covariates were retained in the final model with a significant decrease in the objective function value (dOFV, *P* < 0.001). The final model was evaluated with a goodness-of-fit plot, bootstrap and prediction-corrected visual predictive check (pc-VPC).

### 2.8 Statistical analysis

Continuous variables are expressed as medians and interquartile ranges (IQRs), and categorical variables are expressed as percentages. Unpaired continuous variables were compared using the Mann-Whitney U test, and paired continuous variables were compared using the Wilcoxon test. Kaplan-Meier curves were plotted to determine the overall cumulative percentage of patients who developed ADA. The relationship between the values of ADA-S/N and NAB-B/B0 was analyzed via linear regression. To visually check the relationship between ADA-S/N values and *post hoc* individual estimates of apparent clearance, a restricted cubic spline curve was generated. Differences with a two-tailed *P* value < 0.05 were considered statistically significant. All statistical analysis and graphical figures were performed with GraphPad Prism 8 (La Jolla, CA).

## 3 Results

### 3.1 An integrated immunoassay platform for detecting ADA and NAB against adalimumab

**Figure 1:**
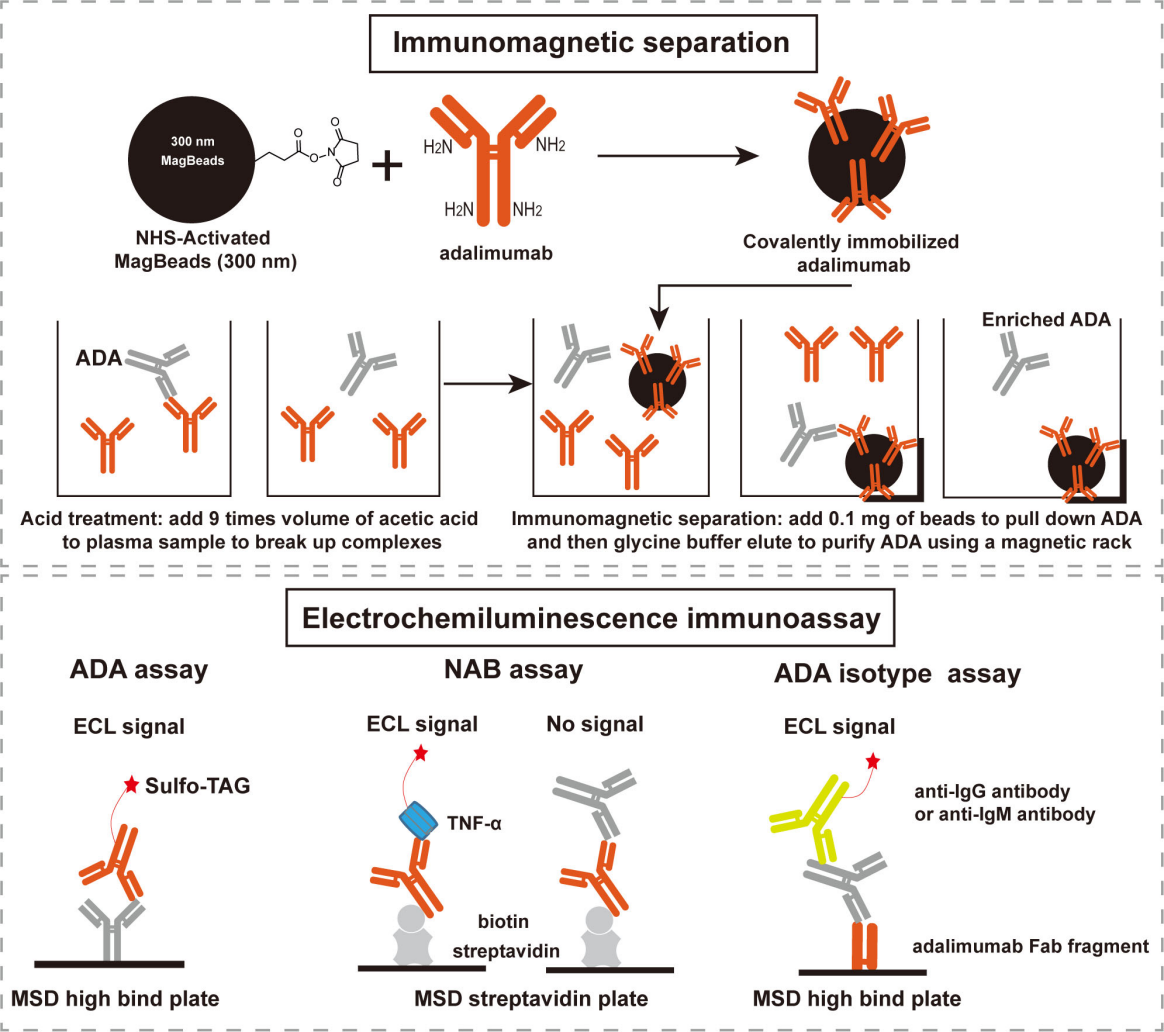
Scheme of the integrated immunoassay platform comprised of immunomagnetic separation and electrochemiluminescence immunoassay. ADA: anti-drug antibodies, NAB: neutralizing antibodies, ECL: electrochemiluminescence, MSD: meso scale discovery.

The schematic diagram and work flow are shown in **Figure 1**. NHS-activated magnetic-beads were utilized to covalently immobilize adalimumab via stable amide linkages formed between NHS and primary amines, thereby enhancing binding capacity and reducing leaching of the immobilized-adalimumab. Subsequently, ADA in the plasma matrix were captured and purified using the functionalized beads, followed by acid dissociation to minimize residual adalimumab from the sample itself. The enriched ADA were then detected using adalimumab labeled with SULFO-TAG after direct coating onto the MSD high-binding plate. The NAB assay was developed using a drug capture and competitive ligand binding format. In the absence of NAB, SULFO-TAG labeled TNF-α binds to the coated biotin adalimumab, resulting in a signal. However, in the presence of NAB, the signal is suppressed. The class of enriched ADA was determined using an indirect ECL immunoassay, where the enriched ADA were captured by the Fab fragment of adalimumab and subsequently detected by specific anti-human IgG or IgM antibody.

**Figure 2:**
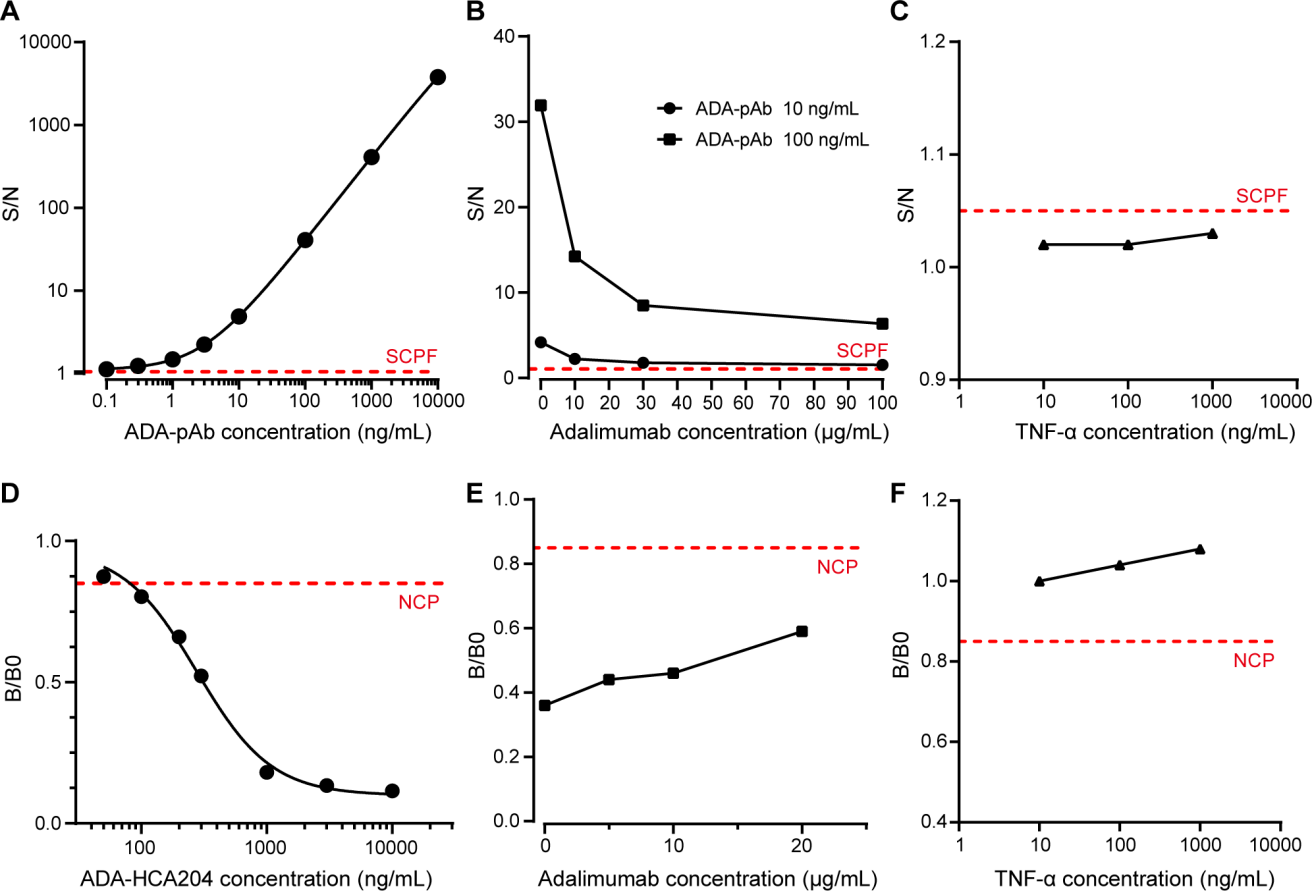
Performance of ADA and NAB assay. **(A)** A typical dose-response curve of ADA-pAb ranging from 0.1 to 10000 ng/mL is generated and fitted by a four-parameter logistic model. **(B)** Positive control samples containing ADA-pAb at 10 ng/mL or 100 ng/mL in the presence of adalimumab at various concentrations (0, 10, 30 and 100 μg/mL) were evaluated in the ADA screening assay. **(C)** Blank plasma matrix with TNF-α at concentrations ranging from 10 to 1000 ng/mL were assessed in the ADA screening assay. **(D)** A typical dose-response curve of ADA-HCA204 ranging from 50 to 10000 ng/mL. The graph is fitted by four-parameter logistic fitting. **(E)** Positive control samples containing ADA-HCA204 at 500 ng/mL in the presence of adalimumab at various concentrations (0, 5, 10 and 20 μg/mL) were assessed in the NAB assay. **(F)** Blank plasma matrixes with TNF-α at concentrations ranging from 10 to 1000 ng/mL was assessed in the NAB assay. The red dotted line illustrates the SCPF in A-C and the NCP in **(D–F)**. ADA, anti-drug antibodies; NAB, neutralizing antibodies; SCPF, screening cut point factor; NCP, neutralizing cut point.

To reveal the detection performance of ADA assay, a panel of 51 adalimumab naïve samples was used to establish the screen cut point factor (SCPF, absence of spiked adalimumab) and confirmatory cut point (CCP, presence of spiked adalimumab) (16). From the 153 values obtained from the 51 individual samples determined three times, a sample was deemed positive if the S/N value from the screening assay exceeded 1.05 (**Supplementary Figure S1A**), and the percent inhibition by the spiked adalimumab in the confirmatory assay was above 10.6% (**Supplementary Figure S1B**). The assay sensitivity was calculated to be 0.4 ng/mL for surrogate pAb in the neat matrix based on the assay’s cut point (**Figure 2A**). Drug-tolerance was assessed by using pAb at concentrations of 10 ng/mL and 100 ng/mL in the presence of adalimumab at varying concentrations (0, 10, 30, and 100 μg/mL). The signal decreased as the adalimumab increased concentration up to 100 μg/mL but remained above the SCPF (**Figure 2B**), indicating a drug-tolerance above 100 μg/mL at 10 ng/mL pAb (drug-tolerance: adalimumab:ADA=10,000:1). Furthermore, TNF-α spiked in the NC at concentrations up to 1000 ng/mL showed no interference (**Figure 2C**).

For validation of the NAB assay, the neutralizing cut point (NCP) was determined to be 0.85 (**Supplementary Figure S1C**), and the sensitivity of the surrogate HCA204 in the neat matrix was found to be 100 ng/mL (**Figure 2D**). The signal of PC sample at 500 ng/mL HCA204 remained below the NCP even with increasing adalimumab concentrations up to 20 μg/mL (**Figure 2E**). Additionally, spiking the NC sample with the target at concentrations up to 1000 ng/mL did not impact the results of the NAB assay (**Figure 2F**). A summary of the key parameters validated for assessing ADA and NAB against adalimumab in human plasma is presented in **Supplementary Table S1**. Therefore, the integrated immunoassay platform consisting of immunomagnetic separation and ECL technique demonstrates high sensitivity and high drug-tolerance, making it suitable for ADA and NAB assessment.

### 3.2 Longitudinal profiles of ADA and NAB against adalimumab

**Figure 3:**
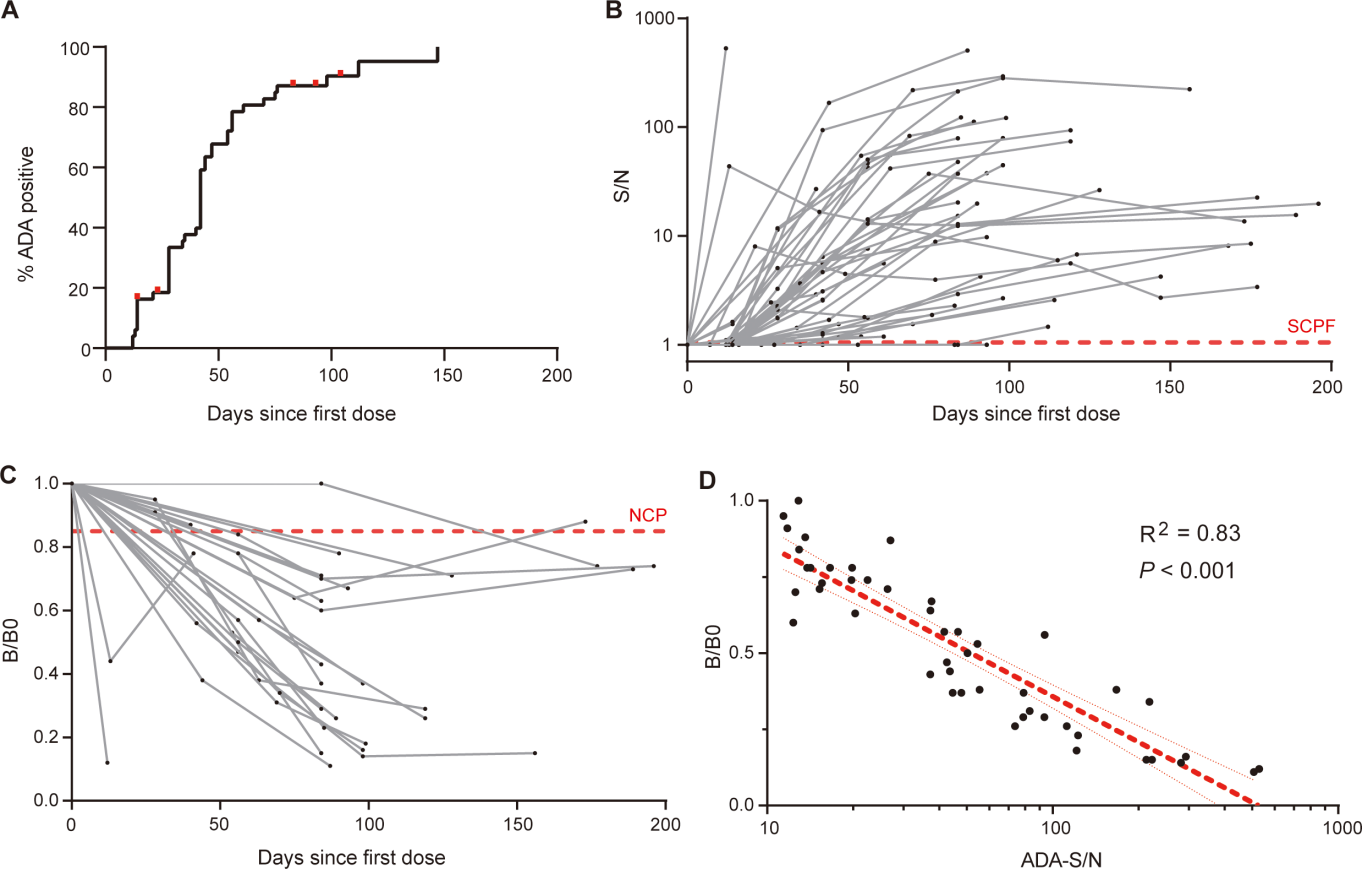
Dynamic profiles of ADA and NAB against adalimumab. **(A)** The overall cumulative percentage of patients who developed ADA during follow-up, with red dots representing censored data for five ADA-negative patients. **(B)** The kinetics of ADA response over treatment time in individual patients, measured by the signal-to-NC ratio (S/N), with the red dotted line indicating the screening cut point factor. **(C)** Kinetics of the NAB response over treatment time in individual patients. The sample signal relative to the blank signal (B/B0) reflects the neutralization capacity, and the red dotted line illustrates the neutralizing cut point. **(D)** There was a strong correlation between ADA levels (S/N) and neutralization activity (B/B0). The red line indicates the linear regression and 95% confidence intervals.

Having defined the sensitivity and drug-tolerance of ADA and NAB assays, we proceeded to examine their kinetic profiles. A longitudinal cohort comprising of 49 patients with ankylosing spondylitis who were initially treated with adalimumab in a real-life clinical setting was analyzed (**Supplementary Table S2**). A total of 201 plasma samples were examined, with no presence of ADA detected in samples collected prior to adalimumab treatment. The overall cumulative percentage of patients testing ADA-positive showed that 90% (44 out of 49) developed ADA during the follow-up period (**Figure 3A**). Out of the five patients who remained ADA-negative, two were lost to follow-up on days 14 and 23, respectively. The ADA-S/N generally increased over time in the early phase and subsequently reached a relative plateau in late phase, with notable variation observed among patients (**Figure 3B**). The enlarged section highlighting early phase showed that 7 out of 43 patients developed ADA after first dosage, and 73% (11/15) of patients developed ADA after second dosage (**Supplementary Figure S2A**). The late response period was observed in eight patients (**Supplementary Figure S2B**), showing relatively flat response. It is noteworthy that all ADA responses were persistent and no transient response was observed. When referring to the sensitivity threshold (100 ng/mL) required by regulatory agency (17) (ADA-S/N=40 based on the ADA calibration curve), only one-third of patients were considered positive (**Supplementary Figure S3**).

Due to the sensitivity considerations, samples with ADA-S/N value exceeding 10 underwent further neutralizing activity assessment, containing 47 samples from 27 patients. Our results revealed that the neutralization capacity and ADA levels displayed similar kinetic profiles (**Figure 3C**). Almost all patients (26 out of 27, 96%) tested positive for NAB during follow-up, with one exception likely attributed to the short duration of follow-up (40 days since the initial dose). We observed a strong correlation between the ADA levels and neutralizing capacity (R^2^ = 0.83, *P* < 0.001, **Figure 3D**), indicating that ADA against adalimumab primarily possess neutralizing properties. Overall, our in-house ADA and NAB assays shed light on the kinetics of ADA and NAB against adalimumab, following the nature of human immune system.

### 3.3 Adalimumab elicits ADA in a T-cell dependent manner

**Figure 4:**
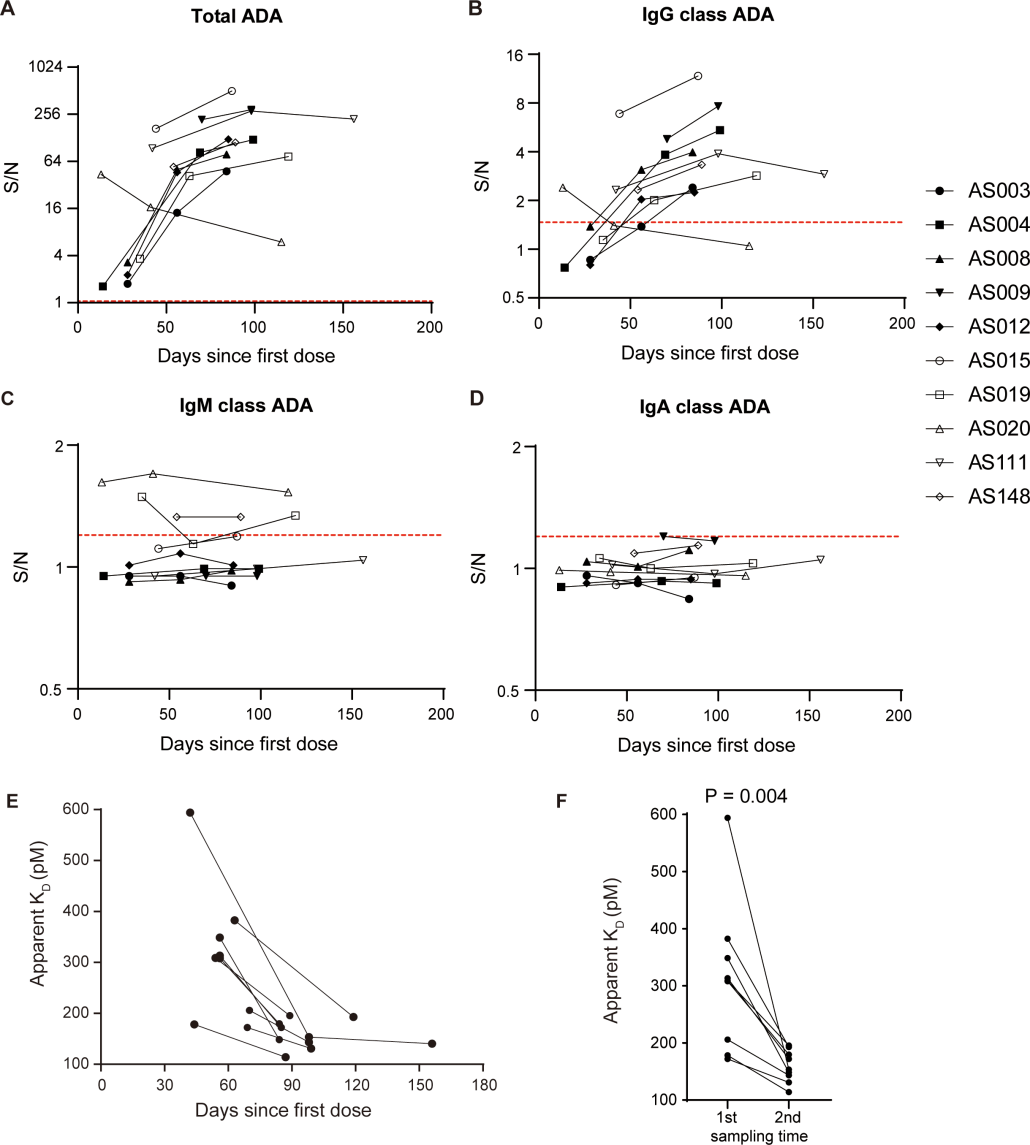
Adalimumab elicits ADA formation in T-cell dependent manner. Profiles of total ADA **(A)**, IgG class ADA **(B)**, IgM class ADA **(C)**, and IgA class ADA **(D)** in ten patients over the course of treatment. The red dotted lines illustrate the corresponding cut points. **(E)** The apparent K_D_ showed a decreasing trend with treatment time in a subset of 9 patients, suggesting an increase in affinity over time. **(F)** The apparent K_D_ values significantly decreased with time extension, as evidenced by the comparison between the first sample and the second sample (median 308.8 vs. 153.3, Wilcoxon test, *P* = 0.004).

In addition to evaluating the onset, duration and neutralizing activity of ADA response, other characteristics of ADA, such as class-switch recombination and affinity maturation, can offer further insights into antibody response in a T-cell dependent or T-cell independent manner (18). We observed a similar kinetic profile between IgG class and total ADA in the ten patients (**Figures 4A, B**). IgM class signals with no increasing trend were observed only in a minority of the samples (**Figure 4C**), and IgA class signals were relatively negative in all tested samples (**Figure 4D**). Profiles of total ADA, IgG class, and IgM class ADA in individual patients were shown in **Supplementary Figure S4**, suggesting that the IgG class is the most prevalent type of ADA against adalimumab.

ADA signals of immunoassays are dependent on both antibody affinity and concentrations (19). We have developed an ECL-based solution equilibrium titration method for apparent affinity estimation of enriched ADA. Our observations in a representative patient showed that the signals of unbound ADA decreased gradually as the concentrations of biotin-adalimumab increasing (**Supplementary Figure S5**), reflecting the principle of solution-phase equilibrium binding interaction. The apparent K_D_ values showed a decreasing trend over time in a subgroup of 9 patients (**Figure 4E**), suggesting an enhancement in affinity with longer treatment duration. Statistical analysis revealed a significant decrease in apparent K_D_ values with prolonged treatment time (*P* = 0.004, **Figure 4F**), supporting the concept of a maturing immune response. In conclusion, the primary mechanism underlying the formation of ADA against adalimumab involves T-cell dependent B cell activation, including class switching from IgM to IgG and the production of antibodies with higher affinity.

### 3.4 Clinical relevance of ADA against adalimumab

After revealing the characteristics of ADA toward adalimumab, we focused on exploring its clinical relevance with adalimumab concentrations and disease relapse. The plasma adalimumab concentrations were measured using a validated sandwich ELISA method. The lower limit of quantification was 62.5 ng/mL, and standard curve fitting with a four-parameter curve ranged from 62.5–2000 ng/mL (**Supplementary Figure S6**). The intra-assay and inter-assay coefficients of variation were ≤6% and ≤10%, respectively (**Supplementary Table S3**).

**Figure 5:**
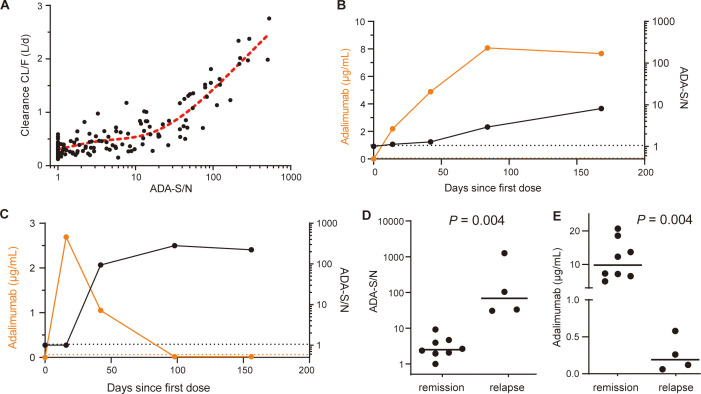
Clinical relevance of ADA against adalimumab. **(A)** Relationship between ADA-S/N values and *post hoc* individual estimates of apparent clearance (CL/F). The red dotted line indicates a restricted cubic spline curve (knots=3). **(B)** The kinetics of adalimumab concentrations (orange dots; left y axis) and ADA-S/N values (black dots; right y axis) in a single representative patient with low ADA levels. The black and orange dotted lines indicate the screening cut point factor of the ADA assay and the lower limit of quantitation of adalimumab, respectively. **(C)** The kinetics of adalimumab concentrations (orange dots; left y axis) and ADA-S/N values (black dots; right y axis) in a single representative patient with high ADA levels. Black and orange dotted lines indicate the screening cut point factor of the ADA assay and the lower limit of quantitation of adalimumab, respectively. **(D)** Median ADA-S/N values were significantly higher for patients at disease relapse than for patients in remission (68.25, n=4 vs. 2.49, n=8, Mann-Whitney test, *P* = 0.004). The black lines show the medians. **(E)** Median adalimumab concentrations were significantly lower for patients at disease relapse than for patients in remission (0.19 μg/mL vs. 9.8 μg/mL, Mann-Whitney test, *P* = 0.004). The black lines show the medians.

A population pharmacokinetic model was established to quantitatively investigate the impact of ADA levels on adalimumab pharmacokinetics using data from a longitudinal cohort of 49 patients. The parameters and evaluations of the model are presented in **Supplementary Figures S7, S8**. The addition of ADA levels into the final model reduced interindividual variability in clearance (CL/F) from 53.5% to 35.4% (**Supplementary Table S4**). A scatter plot illustrated a positive correlation between model-estimated individual clearance and observed ADA-S/N values (**Figure 5A**), suggesting that an ADA-S/N > 10 could be a noteworthy threshold affecting adalimumab exposure based on visual inspection. **Figures 5B, C** demonstrate that adalimumab trough concentration initially rose post-administration in the absence of ADA formation, with levels stabilizing in a patient with low ADA levels (**Figure 5B**). In contrast, a patient with high ADA levels experienced a decline in adalimumab trough levels (**Figure 5C**).

To further investigate the relationship between ADA levels and disease relapse, we conducted a retrospective analysis using data from 12 patients (**Supplementary Table S5**). The median ADA-S/N values were found to be higher in patients who experienced disease relapse compared to those who maintained remission (68.25, n=4 vs. 2.49, n=8, *P* = 0.004, **Figure 5D**). Notably, there was no overlap between the two groups. Furthermore, the adalimumab concentrations were considerably lower in patients with disease relapse compared to those in remission (median 0.19 μg/mL vs. 9.8 μg/mL, *P* = 0.004, **Figure 5E**). Based on these findings regarding ADA levels, drug exposure and disease relapse, it is postulated that an ADA-S/N > 10 could potentially be a clinically relevant threshold.

## 4 Discussion

Here, a highly sensitive and drug-tolerant immunoassay platform was developed for evaluating immunogenicity against adalimumab. This platform involved an affinity separation procedure using drug-specific covalently coupled magnetic beads combined with the MSD-ECL system. The method allowed us to uncover the broad complexity of ADA response against adalimumab, such as kinetics profile, neutralizing capacity, class switching, and affinity maturation. The study also highlighted the implications of these findings on the drug’s pharmacokinetics and effectiveness, proposing a potentially clinically meaningful threshold for clinical applications.

The innovative analytical platform has demonstrated exceptional performance and shows promising as a versatile analytical protocol for evaluating the immunogenicity to biopharmaceuticals. Compared to our previous method involving biotin-drug extraction and acid dissociation (20), the preparation of beads covalently coupled with adalimumab was found to be crucial in minimizing drug carryover during sample treatment, thereby preventing biotin-drug leaching from streptavidin beads (21). Particularly, the magnetic bead separation procedure plays a vital role in achieving drug-tolerance of at least 20 μg/mL for assessing NAB using competitive immunoassay, as drug carryover significantly impacts the subsequent NAB assay (22, 23). Furthermore, MSD-ECL technology offers highly sensitive and robust assays, enhancing sensitivity down to 0.4 ng/mL and broadening the dynamic range up to 10000 ng/mL for ADA assay (24). Advanced technology provides a strong foundation for assessing ADA magnitude with a suitable signal to noise ratio (25). Notably, ADA enrichment using magnetic bead separation is a simpler strategy compared to drug removal methods in reducing residual drug interference (26). The overall workflow can be carried out in a semi-automated manner using a 96-well microtiter plate.

Utilizing the integrated analytical platform, we successfully elucidated the comprehensive profiles of ADA against adalimumab, such as onset time, duration, kinetics, class-switching and affinity maturation. These findings suggest that existing information on immunogenicity against adalimumab from development and early studies may now be outdated. A critical next step lies in translating these findings into clinical practice. Our novel immunoassay revealed that approximately 90% of the patients will persistently develop ADA against adalimumab, representing one of the highest incidences reported in literatures (7, 27). Our observations revealed a distinct pattern in ADA formation kinetics, reminiscent of the kinetic view in adaptive immune responses to vaccines or foreign antigens (28, 29). This pattern is characterized by an initial phase of ADA production within 2–4 weeks, followed by a maturation of the immune response over approximately 3 months. Significant variation were noted in ADA magnitude among individuals, with the underlying mechanism still elusive. Notably, our study emphasizes the importance of monitoring individual ADA dynamics rather than focusing solely on absolute concentrations, as this idea may offer valuable predictive information regarding the potential loss-of-response to infliximab treatment (30). Our data also suggest that proactive monitoring of ADA formation could aid in early prediction of treatment response issues prior to the occurrence of clinical symptoms (31). However, the clinical implications of this strategy warrant further investigation. Additionally, our findings indicate that all patients were persistently positive according to the supersensitive assay, leading us to speculate that transient ADA may be caused by less sensitive assays (31). It is generally believed that transient ADA, which are typically present at low titers, may not significantly impact treatment efficacy (20, 32). Furthermore, our observations suggest that the humoral response to biopharmaceuticals resembles that of a vaccine-like immune response, with repeated administrations potentially eliciting a stronger immune response akin to booster vaccines (33). The development of high-affinity IgG class antibodies following repeated adalimumab dosing indicates T-cell dependent immune response dominance. Conversely, an extrafollicular T-cell independent immune response was noted following initial infliximab administration (34). It is important to consider that alterations in antibody affinity over time could affect results when relying solely on the quantification of enriched ADA masses through LC-MS/MS techniques (35). While the format in which ADA are enriched by protein-A beads and detected by the Fab fragment of adalimumab may be an alternative method (36).

The strong correlation between the ADA-S/N and the neutralization capacity of NAB suggests that the ADA response to adalimumab could be characterized as anti-idiotype responses. This indicates that nearly all ADA are anti-idiotype antibodies and NAB under the conditions of the supersensitive assay. Previous studies using cell-based or immunoassays had limited sensitivity, resulting in only a minority of ADA being tested as NAB and complicating the interpretation of NAB results (37). Patient-derived monoclonal antibodies in studies involving the antibody repertoire of ADA-positive patients showed a restricted response, with all ADA competing for TNF-α binding (38, 39). In addition, ADA epitopes were identified mostly located in the adalimumab variable region by epitope mapping assay via peptide microarray (40). The presence of ADA leads to suboptimal drug exposure and treatment response by increasing drug clearance and blocking the pharmacological effect of adalimumab. Therefore, assessing NAB results does not seem to provide additional value compared to the more readily available ADA results.

Establishing a clinically meaningful threshold relevant to adalimumab pharmacokinetics and clinical efficacy is crucial for guiding clinical practice. Our study propose a provisional threshold of ADA-S/N above 10, showing correlation with disease relapse and lower adalimumab concentrations. When high ADA levels are detected, patients should receive increased attention in terms of treatment decisions, potentially including the addition of immune-modulators or adjustments to dosing intervals. It is important to note that different bioanalysis methods may yield varying results, typically of a qualitative nature. Although ADA-S/N value correlates well with titer and could serve as an equivalent (25), single S/N value is highly dependent on the assay and its performance, which pose significant challenges for its harmonization and application in clinical practice.

A limitation of the present study is the lack of efficacy data from the longitudinal cohort. The association between ADA levels and treatment relapse was established in a cross-sectional cohort with a small sample size. Notably, the absence of overlap in ADA levels between the relapse and remission groups strengthened the validity of the findings. Therefore, the clinical significance of the provisional threshold based on a limited data set requires additional validation and refinement through well-designed prospective trials. Additionally, the limited sensitivity of NAB assay in comparison to ADA assay prevented the detection of samples with low ADA levels. Fortunately, the NAB assay can cover the samples exceeding the clinical significance threshold (ADA-S/N>10). Moreover, enriched ADA were not typed to subclasses of IgG, which can be performed using specific antibodies if necessary.

Collectively, our study presented an integrated immunoassay platform that combines immunomagnetic separation and ECL technique, tailored for evaluating immunogenicity against adalimumab. By utilizing the highly sensitive and drug-tolerant assays, we were able to comprehensively outline the characteristics of ADA against adalimumab, offering fresh insights into immunogenicity and its clinical implications. Future clinical trials will be essential to determine whether proactive monitoring of ADA levels and drug concentrations is correlated with favorable outcomes.

## Data Availability

The original contributions presented in the study are included in the article/**Supplementary Material**. Further inquiries can be directed to the corresponding authors.
