# Supplementary material for: Dynamics and implications of anti-drug antibodies against adalimumab using ultra-sensitive and highly drug-tolerant assays
Source: Front Immunol. 2024 Aug 22;15:1429544. doi: 10.3389/fimmu.2024.1429544 (PMC11374634; doi:10.3389/fimmu.2024.1429544)
Supplement: Supplementary file 1 [file DataSheet1.docx]

***Supplemental materials***

**Dynamics and implications of anti-drug antibodies against adalimumab using ultra-sensitive and highly drug-tolerant assays**

**Supplementary Figure S1.** Scatter plots of ADA-S/N values (A, screening data), inhibition percent by spiked drug (B, confirmatory data) and NAB-B/B0 values (C, neutralizing data) for individual plasma data from 51 patients with ankylosing spondylitis tested three times. Red lines indicate cut points, red dots indicate outliers. *SCPF* screening cut point factor. SCPF is determined by non-parametric 95^th^ percentile based on S/N values, after exclusion of outliers. *CCP* confirmatory cut point. CCP is determined by non-parametric 99^th^ percentile based on inhibition percent values, after exclusion of outliers. *NCP* neutralizing cut point. NCP is determined by non-parametric 99^th^ percentile based on B/B0 values.

**Supplementary Figure S2**. The partial enlarged views of Figure 3B. The kinetics of ADA response over treatment time in individual patients within a follow-up time of 50 days (A), and between 80 days to end (B).

**Supplementary Figure S3**. The kinetics of ADA response over treatment time in individual patients when detection sensitivity was set at 100 ng/mL (ADA-S/N=40), measured by the signal-to-NC ratio (S/N).

**Supplementary Figure S4**. Profiles of total ADA, IgG class ADA, and IgM class ADA over the course of treatment in individual patient.

**Supplementary Figure S5.** Fitted affinity curves of 3 samples collected at day 42, day 98 and day 156 since first adalimumab administration from one representative patient.

**Supplementary Figure S6.** Typical standard curve adalimumab sandwich ELISA. The graph was constructed using the adalimumab concentration, ranging from 31.25 – 2000 ng/mL, and optical dencity (450 nm – 630 nm) by four-parametric logistic fitting.

**Supplementary Figure S7.** Goodness-of-fit plots of the final adalimumab pharmacokinetics model. (A) Observed versus population predicted concentrations. (B) Observed versus individual predicted concentrations. (C) Conditional weighted residuals versus population predicted concentrations. (D) Conditional weighted residuals versus time.

**Supplementary Figure S8.** Prediction-corrected visual predictive check (pc-VPC) of adalimumab concentrations in the final model.

**Supplementary Table S1.** Summary of key parameters validated for assessment of ADA and NAB against in human plasma.

**Supplementary Table S2.** Demographics of longitudinal cohort.

**Supplementary Table S3**. Intra- and inter-assay precision and accuracy of quality controls (n=6).

**Supplementary Table S4**. Population pharmacokinetic parameters of adalimumab and bootstrap results.

**Supplementary Table S5**. Individual data of cross-sectional cohort.

**
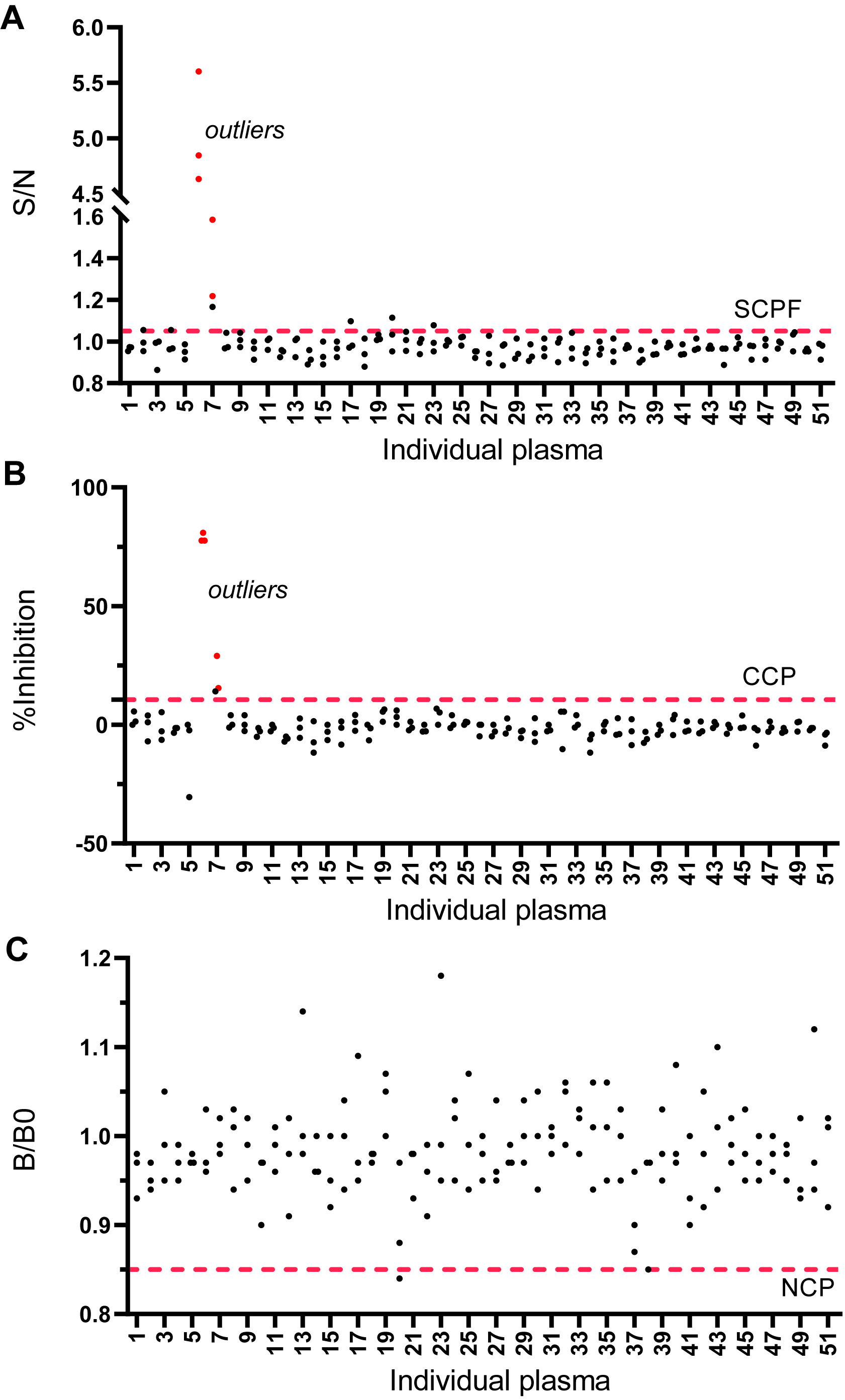
**

**Supplementary Figure S1.** Scatter plots of ADA-S/N values (A, screening data), inhibition percent by spiked drug (B, confirmatory data) and NAB-B/B0 values (C, neutralizing data) for individual plasma data from 51 patients with ankylosing spondylitis tested three times. Red lines indicate cut points, red dots indicate outliers which are identified using Tukey’s outlier criteria with k=3. *SCPF* screening cut point factor. SCPF is determined by non-parametric 95^th^ percentile based on S/N values, after exclusion of outliers. *CCP* confirmatory cut point. CCP is determined by non-parametric 99^th^ percentile based on inhibition percent values, after exclusion of outliers. *NCP* neutralizing cut point. NCP is determined by non-parametric 99^th^ percentile based on B/B0 values.


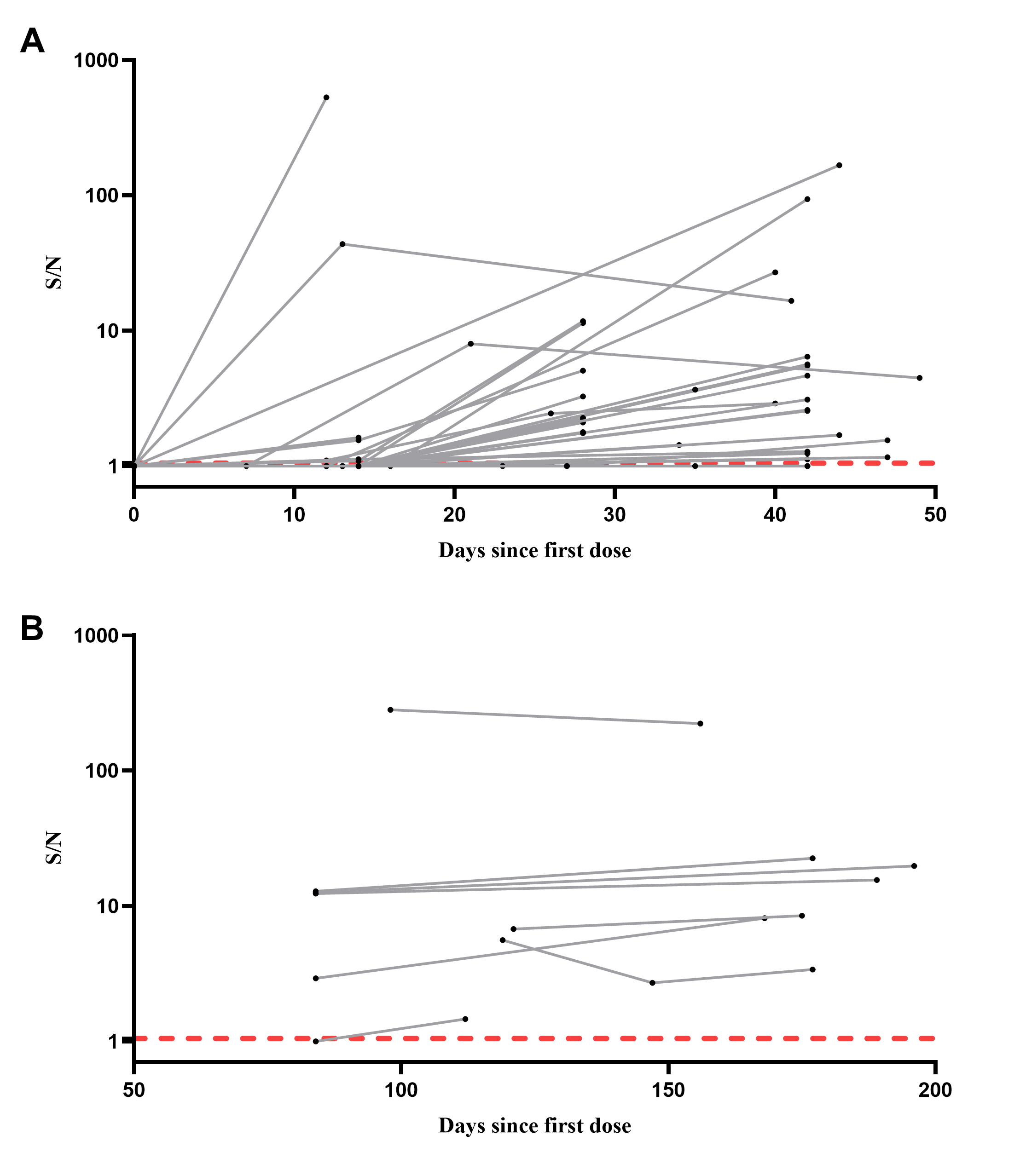


**Supplementary Figure S2**. The partial enlarged views of Figure 3B. The kinetics of ADA response over treatment time in individual patients within a follow-up time of 50 days (A), and between 80 days to end (B).

**
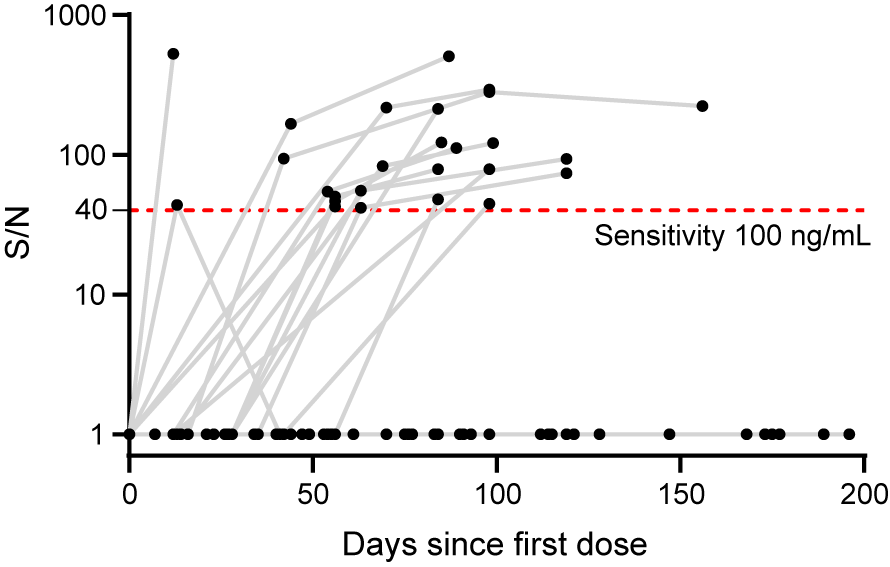
**

**Supplementary Figure S3**. The kinetics of ADA response over treatment time in individual patients when detection sensitivity was set at 100 ng/mL (ADA-S/N=40), measured by the signal-to-NC ratio (S/N).


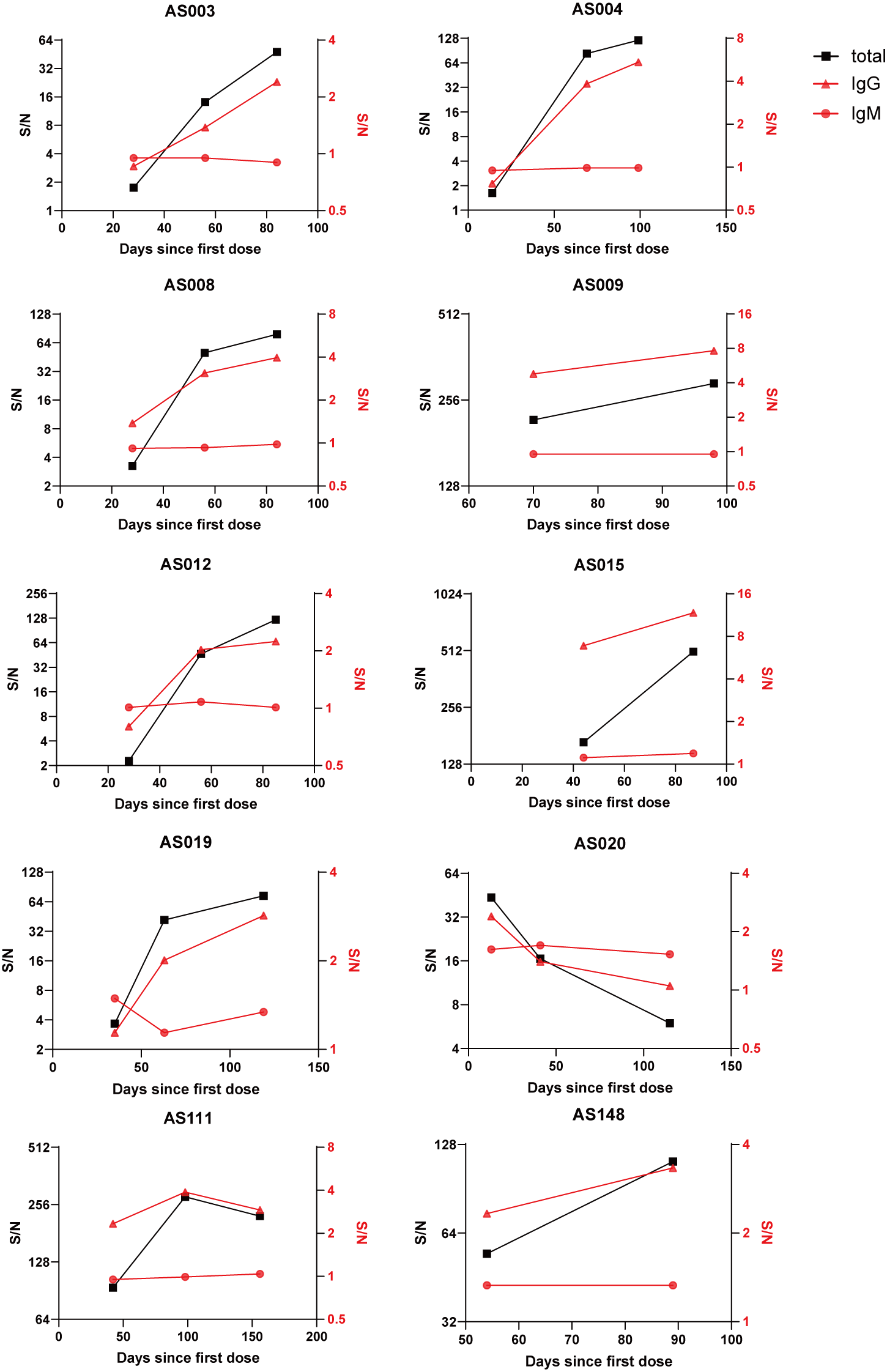


**Supplementary Figure S4**. Profiles of total ADA, IgG class ADA, and IgM class ADA over the course of treatment in individual patient.


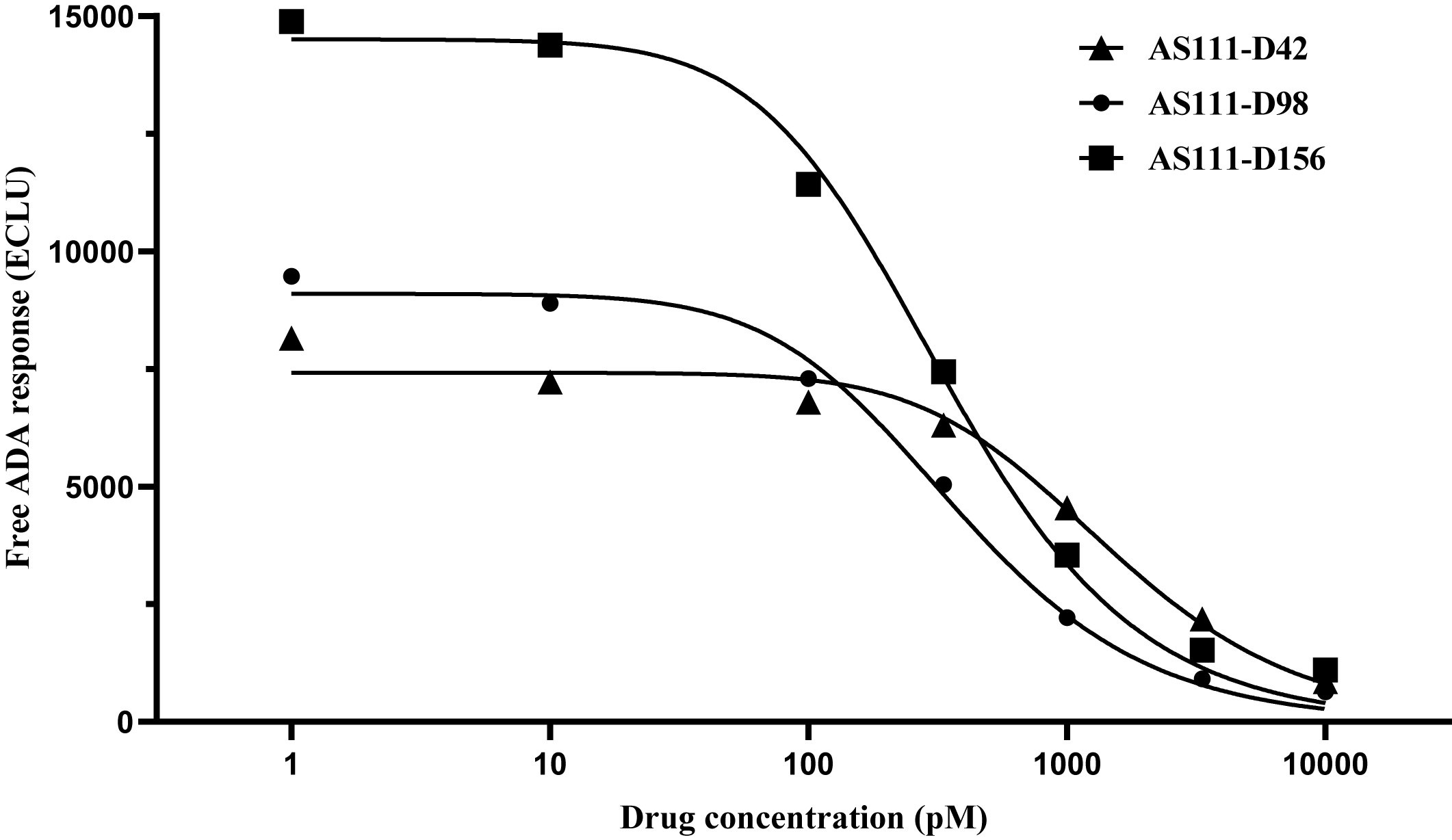


**Supplementary Figure S5.** Fitted affinity curves of 3 samples collected at day 42, day 98 and day 156 since first adalimumab administration from one representative patient.


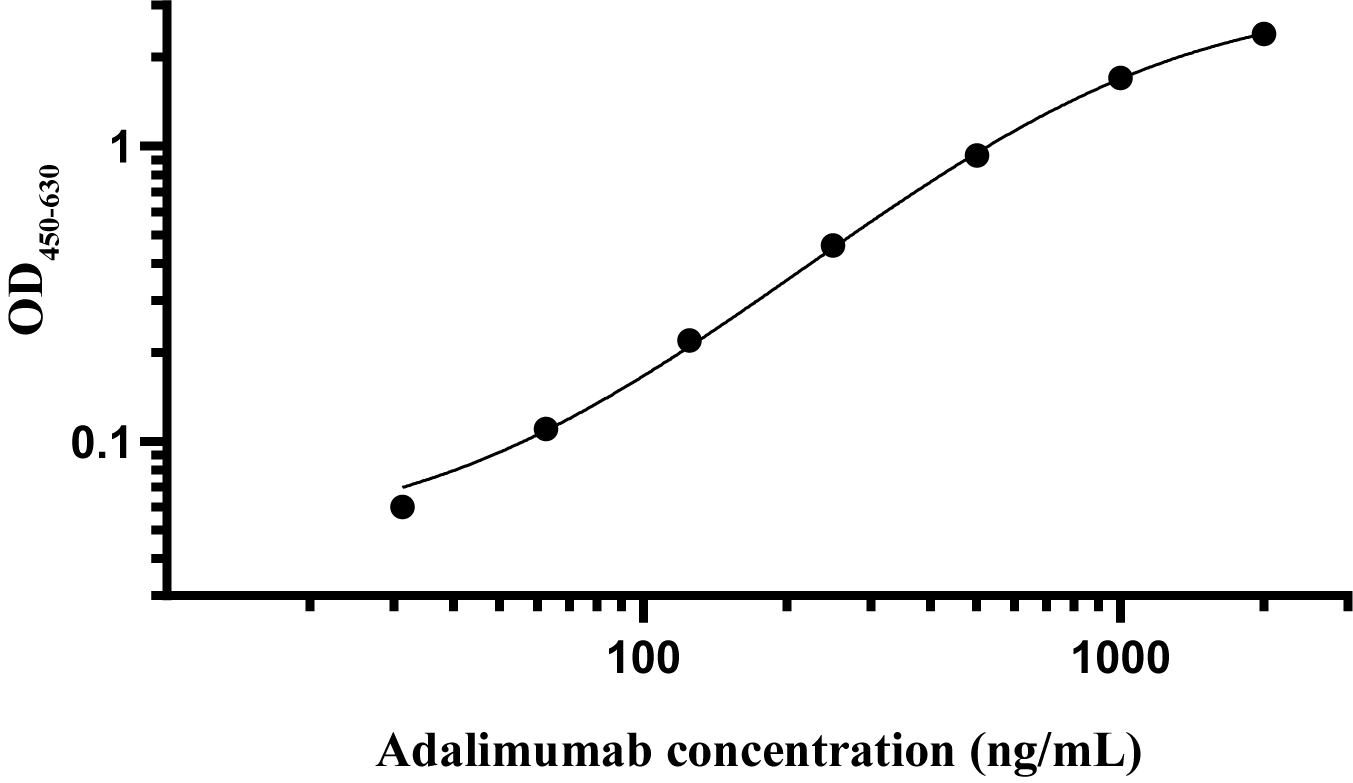


**Supplementary Figure S6.** Typical standard curve adalimumab sandwich ELISA. The graph was constructed using the adalimumab concentration, ranging from 31.25 – 2000 ng/mL, and optical dencity (450 nm – 630 nm) by four-parametric logistic fitting.


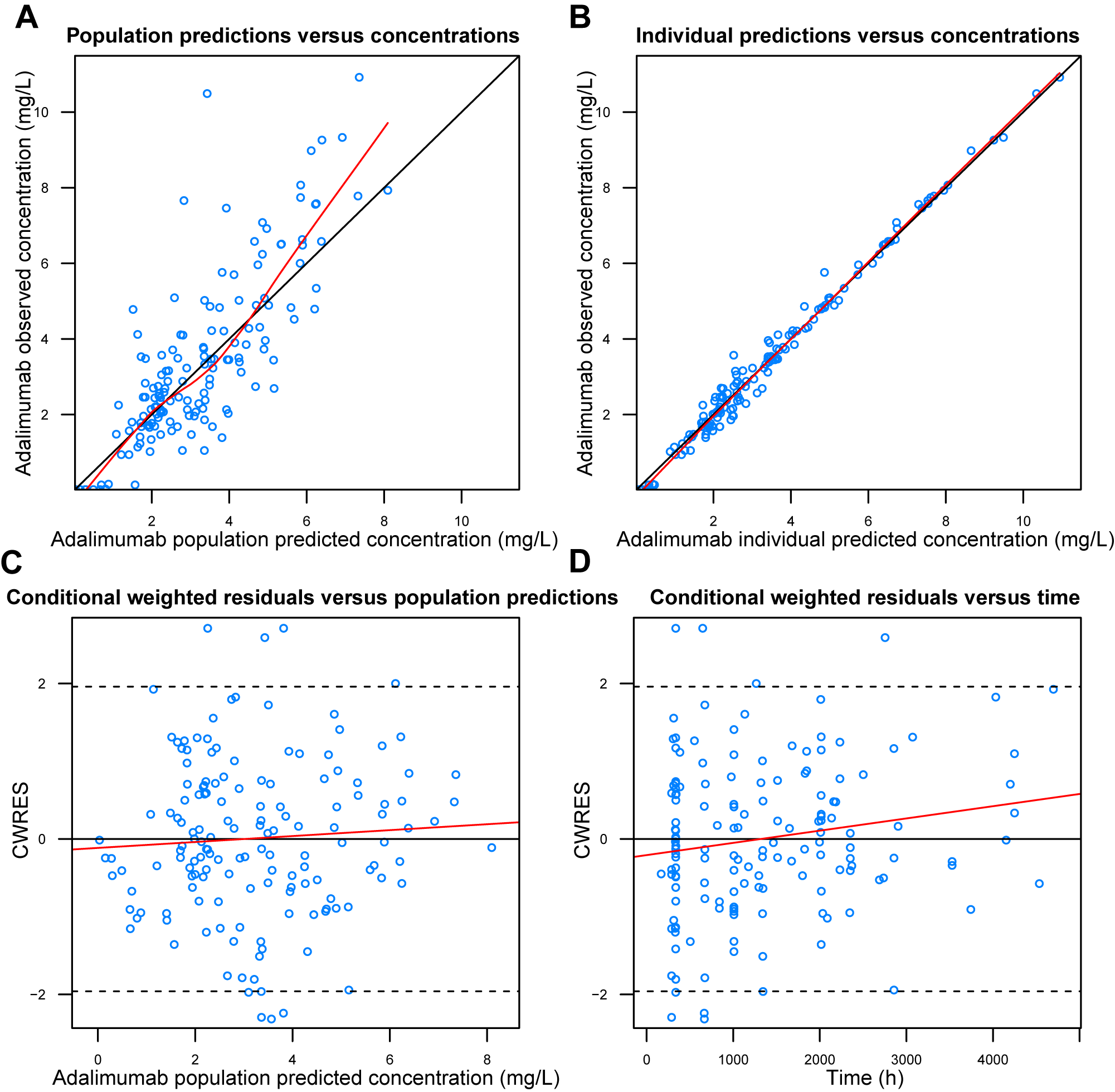


**Supplementary Figure S7.** Goodness-of-fit plots of the final adalimumab pharmacokinetics model. (A) Observed versus population predicted concentrations. (B) Observed versus individual predicted concentrations. (C) Conditional weighted residuals versus population predicted concentrations. (D) Conditional weighted residuals versus time.


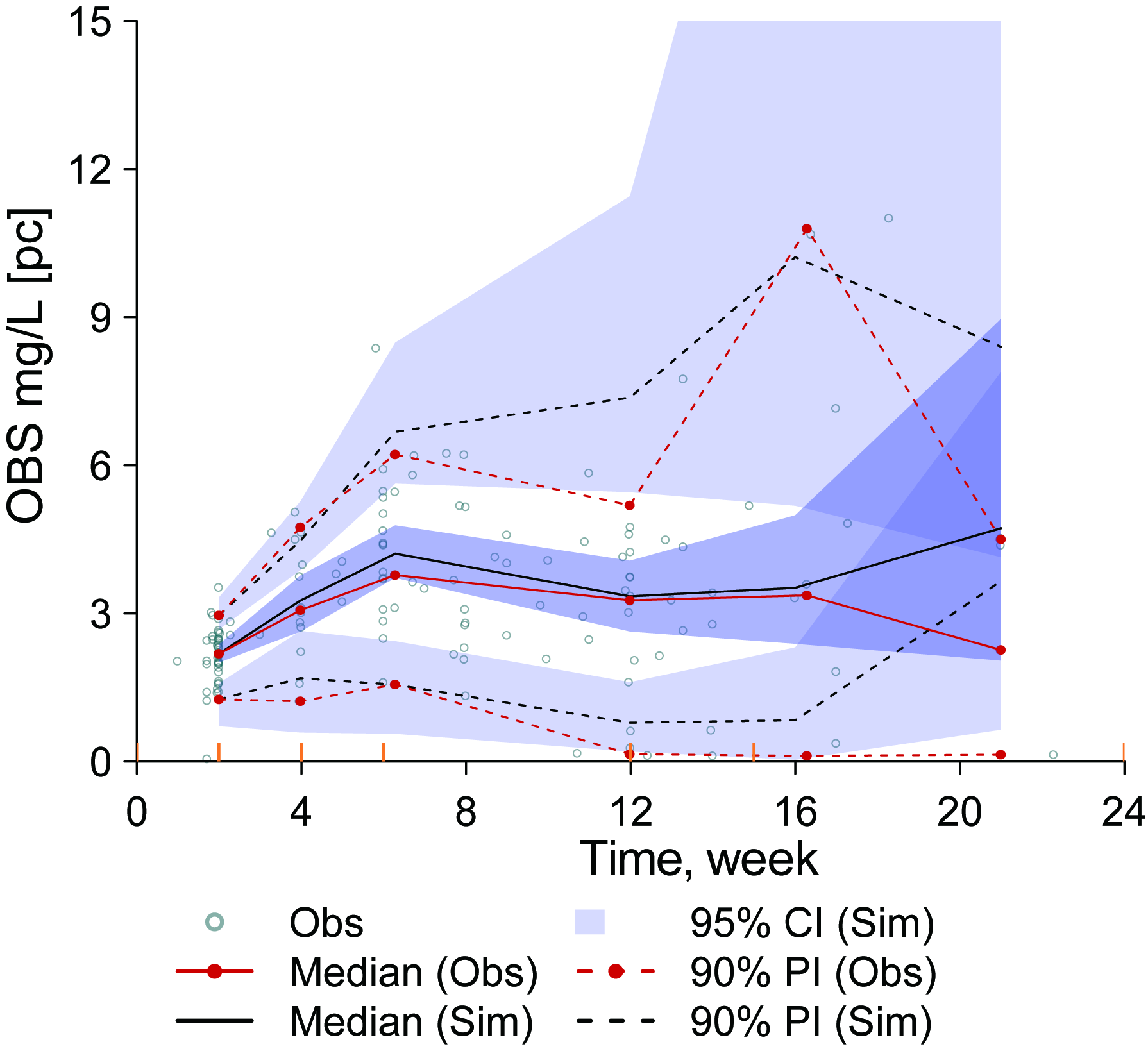


**Supplementary Figure S8.** Prediction-corrected visual predictive check (pc-VPC) of adalimumab concentrations in the final model.

**Supplementary Table S1.** Summary of key parameters validated for assessment of ADA and NAB against in human plasma

| Validation Parameter | Results |
| --- | --- |
| **ADA assay** | |
| Positive control (PC) | rabbit-anti-adalimumab idiotype polyclonal antibodies |
| PC standard range | 0.4 – 10000 ng/mL |
| Assay sensitivity | 0.4 ng/mL |
| Screening cut point factor | S/N 1.05 (5 outliers) |
| Confirmatory drug concentration | 10 μg/mL |
| Confirmatory cut point | % Inhibition 10.6 (5 outliers) |
| Drug tolerance | Tolerant up to 100 μg/mL (PC 10 ng/mL) |
| Target tolerance | Tolerant up to 1000 ng/mL |
| Prozone | Not present up to 10000 ng/mL |
| Low PC | 10 ng/mL |
| High PC | 10000 ng/mL |
| Intra-assay precision (NC, %CV) | Screen 3.12, Confirm 1.26 |
| Intra-assay precision (LPC, %CV) | Screen 4.24, Confirm 2.17 |
| Intra-assay precision (HPC, %CV) | Screen 3.66, Confirm 4.20 |
| Inter-assay precision (NC, %CV) | Screen 4.19, Confirm 6.59 |
| Inter-assay precision (LPC, %CV) | Screen 7.67, Confirm 5.33 |
| Inter-assay precision (HPC, %CV) | Screen 9.05, Confirm 11.35 |
| Minimum required dilution (MRD) | 1 in 10 |
| Selectivity | No effect of individual or hemolysis plasma |
| **NAB assay** | |
| Neutralizing cut point | B/B0 0.85 (0 outliers) |
| Assay sensitivity | 100 ng/mL |
| Low PC | 300 ng/mL |
| High PC | 10000 ng/mL |
| Intra-assay precision (NC, %CV) | 2.24 |
| Intra-assay precision (LPC, %CV) | 4.49 |
| Intra-assay precision (HPC, %CV) | 3.10 |
| Inter-assay precision (NC, %CV) | 2.73 |
| Inter-assay precision (LPC, %CV) | 4.85 |
| Inter-assay precision (HPC, %CV) | 7.87 |
| Drug tolerance | Tolerant up to 20 μg/mL (PC 500 ng/mL) |
| Target tolerance | Tolerant up to 1000 ng/mL |

**Supplementary Table S2.** Demographics of longitudinal cohort

| Characteristic | All patients (N=49) |
| --- | --- |
| Male, N (%) | 37 (75.5) |
| Age, years, median (IQR) | 37 (30-44) |
| Height, cm, median (IQR) | 170 (165-172) |
| Weight, kg, median (IQR) | 70 (60-75) |

**Supplementary Table S3**. Intra- and inter-assay precision and accuracy of quality controls (n=6)

| Control (ng/mL) | Intra-assay | | |  | Inter-assay | | |
| --- | --- | --- | --- | --- | --- | --- | --- |
|  | Accuracy (RE%) | Precision (CV%) | Total error (\|RE\|%+CV%) |  | Accuracy (RE%) | Precision (CV%) | Total error (\|RE\|%+CV%) |
|  |  |  |  |  |  |  |  |
| 2000 | -2.24 | 5.43 | 7.67 |  | 0.37 | 8.51 | 8.87 |
| 1600 | -1.21 | 1.72 | 2.93 |  | -1.36 | 4.88 | 6.23 |
| 400 | 0.23 | 2.60 | 2.82 |  | -1.07 | 1.84 | 2.90 |
| 160 | -0.57 | 1.57 | 2.14 |  | 0.69 | 3.34 | 4.03 |
| 62.5 | -1.12 | 4.70 | 5.82 |  | -4.64 | 3.79 | 8.43 |

**Supplementary Table S4**. Population pharmacokinetic parameters of adalimumab and bootstrap results

| Parameter | Final model |  | Bootstrap | | |
| --- | --- | --- | --- | --- | --- |
|  | Estimate (RSE%) |  | Median | 95% CI | RSE% |
| CL/F (L/d) | 0.862 (10) |  | 0.874 | 0.703-1.04 | 10.3 |
| V/F (L) | 14.5 (6.8) |  | 14.4 | 12.5-16.4 | 7.2 |
| ADA for CL/F | 0.292 (16.4) |  | 0.296 | 0.187-0.376 | 16.3 |
| IIV_CL/F | 0.354 (13.6) |  | 0.346 | 0.235-0.442 | 15.4 |
| Additive error (mg/L) | 0.410 (21) |  | 0.392 | 0.140-0.548 | 26.7 |

The final model was described as:

$$\mathrm{CL}/F（L/d）=0.862\times\left( \frac{\mathrm{FFM}}{56.1} \right)^{0.75}\times\left( \frac{\mathrm{ADA}}{30} \right)^{0.292}$$

$$V/F（L）=14.5\times\frac{\mathrm{FFM}}{56.1}$$

*FFM* fat free mass, *ADA* ADA-S/N value, *IIV* inter-individual variability.

**Supplementary Table S5**. Individual data of cross-sectional cohort

| Patient # | Disease (duration) | Age,  (year) | Sex | Combination therapy | Disease status | Adalimumab dosing regimen | Drug concentration  (μg/mL) | ADA-S/N |
| --- | --- | --- | --- | --- | --- | --- | --- | --- |
| TDM#1 | AS (9 y) | 27 | male | etoricoxib | relapse | 40 mg once-two weeks dosing for 9 months | 0.58 | 103.39 |
| TDM#2 | CD (12 y) | 43 | male | NA | relapse | 40 mg once-two weeks dosing for 8 months | 0.12 | 33.11 |
| TDM#3 | CD (1 y) | 20 | male | NA | remission | 40 mg once-two weeks dosing for 6 months | 13.69 | 1.00 |
| TDM#4 | CD (3 y) | 32 | female | NA | remission | 40 mg once-two weeks dosing for 12 months | 20.65 | 2.06 |
| TDM#5 | AS (8 y) | 32 | female | NA | relapse | 40 mg once-two weeks dosing for 10 months | 0.06 | 1252.43 |
| TDM#6 | AS (6 y) | 47 | female | NA | remission | 40 mg once-three weeks dosing for 23 months | 7.30 | 9.17 |
| TDM#7 | CD (4 y) | 57 | male | NA | relapse | 40 mg once-two weeks dosing for 10 months | 0.26 | 30.56 |
| TDM#8 | CD (4 y) | 40 | male | azathioprine | remission | 40 mg once-two weeks dosing for 19 months | 6.50 | 2.64 |
| TDM#9 | CD (4 y) | 25 | female | NA | remission | 40 mg once-two weeks dosing for 20 months | 18.57 | 1.94 |
| TDM#10 | AS (8 y) | 66 | female | sulfasalazine | remission | 40 mg once-two weeks dosing for 14 months | 12.30 | 3.91 |
| TDM#11 | CD (3 y) | 55 | female |  | remission | 40 mg once-two weeks dosing for 6 months | 4.95 | 4.66 |
| TDM#12 | AS (10 y)  CD (3 y) | 39 | male |  | remission | 40 mg once-month dosing for 34 months | 7.15 | 2.34 |

Abbreviation: *AS* ankylosing spondylitis, *CD* Crohn’s disease, *y* years.
